# *FLOWERING LOCUS T* mediates photo-thermal timing of inflorescence meristem arrest in *Arabidopsis thaliana*

**DOI:** 10.1093/plphys/kiad163

**Published:** 2023-03-21

**Authors:** Pablo González-Suárez, Catriona H Walker, Tom Bennett

**Affiliations:** School of Biology, Faculty of Biological Sciences, University of Leeds, Leeds LS2 9JT, UK; School of Biology, Faculty of Biological Sciences, University of Leeds, Leeds LS2 9JT, UK; School of Biology, Faculty of Biological Sciences, University of Leeds, Leeds LS2 9JT, UK

## Abstract

Plants integrate environmental information into their developmental program throughout their lifetime. Light and temperature are particularly critical cues for plants to correctly time developmental transitions. Here, we investigated the role of photo-thermal cues in the regulation of the end-of-flowering developmental transition in the model plant Arabidopsis (*Arabidopsis thaliana*). We found that increased day length and higher temperature during flowering promote earlier inflorescence arrest by accelerating the rate at which the inflorescence meristem (IM) initiates floral primordia. Specifically, we show that plants arrest at a photo-thermal threshold and demonstrate that this photo-thermally mediated arrest is mediated by the floral integrator FLOWERING LOCUS T (FT), a known activator of flowering. *FT* expression increased over the duration of flowering, peaking during IM arrest, and we show that this is necessary and sufficient for photo-thermally induced arrest. Our data demonstrate the role of light and temperature, through *FT*, as key regulators of end-of-flowering. Overall, our results have important implications for understanding and modulating the flowering duration of crop species in changing light and temperature conditions in a warming global climate.

## Teaser

Temperature and day length control the duration of flowering in Arabidopsis through the floral integrator FLOWERING LOCUS T.

## Introduction

Plants spend their lifetimes faced with a variety of environmental challenges, which they must adequately respond to in order to survive and leave progeny (Bratzel and Turck 2015). To do so, plants can use external signals to alter the timing or duration of key developmental processes. This is particularly well exemplified by the decision to enter the reproductive phase in flowering plants (“floral transition”), which is an exceptionally well-studied example of a life-history event that is subject to strong environmental regulation. For reproduction to succeed, floral transition must be properly synchronized with favorable environmental conditions, which is particularly important for monocarpic species that reproduce only once in their lifetime, such as Arabidopsis (*Arabidopsis thaliana*). As such, the floral transition is tightly regulated by a variety of both external and internal cues, including plant age and gibberellins, among others (Srikanth and Schmid 2011). The importance of the environment in the regulation of flowering time is clear, and 3 different pathways have been described which integrate environmental signals into the flowering program: the photoperiod, vernalization, and ambient temperature pathways (Song et al. 2015). As clear indicators of the transition from winter-to-spring, day length and temperature are indeed 2 of the most important cues that influence the timing of floral transition. Cumulative light hours and cumulative temperature exposure (“degree days”) promote flowering in Arabidopsis, but only after past exposure to a prolonged cold period (vernalization).

The day length, temperature, and vernalization pathways converge on the regulation of the *FLOWERING LOCUS T* (*FT*) gene, encoding a phosphatidylethanolamine-binding protein (Kardailsky et al. 1999; Nakamura et al. 2014; Romera-Branchat et al. 2014). Although *FT*-independent pathways for flowering are also important, *FT* is considered a central component in the genetic network that integrates environmental information into the “decision” to undergo floral transition (Srikanth and Schmid 2011; Song et al. 2015). Under inductive conditions, *FT* is expressed in phloem companion cells in the leaves, where FT protein is loaded into the phloem stream and transported to the shoot apical meristem (SAM) (Corbesier et al. 2007). In the SAM, FT protein interacts with a bZIP-type transcription factor named FD (Wigge et al. 2005), forming a complex that promotes the expression of genes responsible for the conversion of the vegetative SAM into an inflorescence meristem (IM) that initiates flowers and ultimately leads to the production of seed and fruit (Corbesier et al. 2007; Notaguchi et al. 2008). A variety of upstream genes regulate *FT* in both the photoperiod and ambient temperature pathways. In Arabidopsis (a facultative long-day plant), the induction of *FT* under long days is achieved through an intricate genetic network that controls the expression and protein stability of both FT and its main transcriptional activator, CONSTANS (CO) (reviewed in Turck et al. 2008). A moderately warm temperature also promotes *FT* expression. Although the ambient temperature pathway is much less understood, some of the upstream regulators of *FT* include *SHORT VEGETATIVE PHASE* (*SVP*), *FLOWERING LOCUS C* (*FLC*), and *FLOWERING LOCUS M* (*FLM*), among others (Lee et al. 2007; Capovilla et al. 2015; Capovilla et al. 2017).

While the floral transition is intensively studied in Arabidopsis and many other species (Ausin et al. 2004; Benlloch et al. 2007), the physiological and molecular events that underlie the end of the reproductive phase remain poorly characterized. We recently proposed a conceptual framework for end-of-flowering, describing at least 4 developmental events that might lead to end-of-flowering (González-Suárez et al. 2020). In Arabidopsis, end-of-flowering is the result of individual inflorescences ceasing their development, a process that occurs quasi-synchronously in different inflorescences of the same plant (Ware et al. 2020). This “inflorescence arrest” results from both the IM stopping to produce new floral primordia (“IM arrest”) and previously initiated primordia arresting their development (“floral arrest”) (Walker et al. 2023). Much like floral transition, the inflorescence arrest is an orchestrated event under genetic and hormonal regulation. It is likely that IM arrest is controlled by the meristem identity marker *WUSCHEL* (*WUS*), whose expression is undetectable in arrested IMs (Wuest et al. 2016). This downregulation of *WUS* seems to be mediated, at least partially, by the plant age pathway through a miRNA-dependent mechanism that leads to the inhibition of *APETALA2* (*AP2*), an activator of *WUS* (Balanzà et al. 2018). Recently, auxin (Ware et al. 2020) and cytokinin (Merelo et al. 2022; Walker et al. 2023) were also linked to the regulation of inflorescence arrest in Arabidopsis. Given the key role of environmental stimuli in plant life-history transitions, it has been hypothesized that environmental signals may also exert control over inflorescence arrest (González-Suárez et al. 2020; Miryeganeh 2021).

Here, our aim was to assess the role of light and temperature in the regulation of inflorescence arrest. Although previous works have reported the response of yield-related traits to temperature (Ibañez et al. 2017), its specific effect on the late development of individual inflorescences has not been reported. Because of this, our focus was to understand light and temperature regulation of inflorescence development, and specifically of IM arrest. Since, as discussed above, photo-thermal cues have well-characterized effects on the timing of floral transition, we hypothesized that light and temperature exposure would also play a key role in controlling the timing of inflorescence arrest and IM arrest in Arabidopsis.

## Results

### Day length and temperature control inflorescence duration

**Figure 1.**
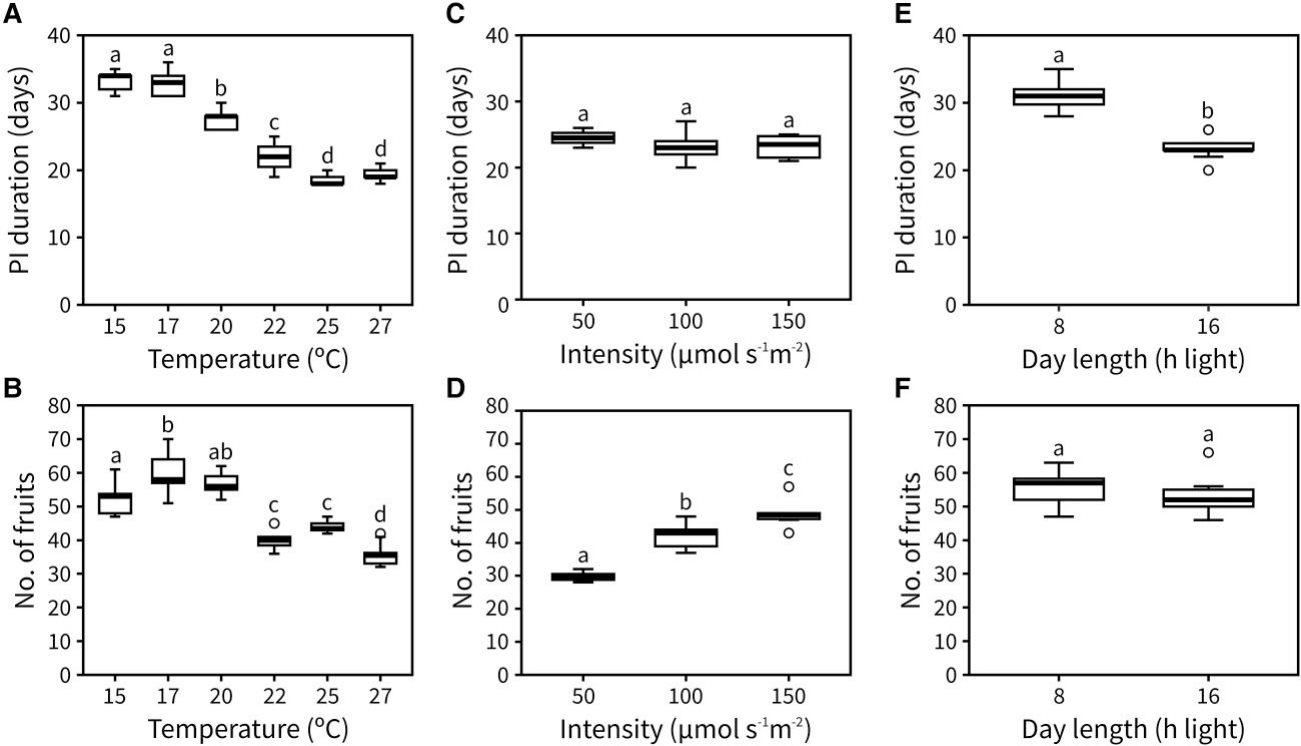
Inflorescence lifespan and fruit set are affected by photo-thermal signals. **A and B)** Duration **(A)** and the number of fruits **(B)** of the PI in plants grown at 15 °C, 17 °C, 20 °C, 22 °C, 25 °C, or 27 °C (*n* = 9 to 12). **C and D)** Duration **(C)** and the number of fruits **(D)** of the PI in plants grown at 50, 100, and 150 *μ*mol s^−1^ m^−2^ (*n* = 4 to 9). **E and F)** Duration **(E)** and the number of fruits **(F)** of the PI in plants grown at 8 and 16 h of day length (*n* = 11 to 12). Different letters indicate statistical differences between conditions (for A–D: ANOVA, Tukey Honestly Significant Difference (HSD), *P* < 0.05, for E and F: Student's *t*-test, *P* < 0.05). Boxes indicate the interquartile range. The central line indicates the median, and whiskers show minimum and maximum values.

To understand the role of temperature in the end of flowering, we recorded the time between visible bolting and inflorescence arrest of the primary inflorescence (hereafter “PI duration”) and the number of fruits produced at 6 different ambient temperatures: 15 °C, 17 °C, 20 °C, 22 °C, 25 °C, and 27 °C. The day length was kept at 16 h light and the light intensity at 100 *μ*mol s^−1^ m^−2^ for all temperatures tested. Under our growth conditions, temperature showed a clear effect on PI duration, which was shorter at warmer conditions (Fig. 1A). The number of fruits was also affected by temperature, with a maximum number achieved between 17 °C and 20 °C (Fig. 1B). The observed phenotypes may partially result from a general acceleration of growth under warmer temperatures. In agreement with this, the average rate of flower opening (i.e. florochron) was higher the warmer the temperature (Supplemental Fig. S1).

To understand the role of light in the end of flowering, we tested the effect of light intensity and day length on PI duration and the number of fruits produced by growing plants at 3 different intensities (50, 100, and 150 *μ*mol s^−1^ m^−2^; all at 16 h of day length) and 2 different day lengths (8 and 16 h light; both at 100 *μ*mol s^−1^ m^−2^), respectively. The temperature was kept at 22 °C for all conditions tested. We did not find any statistical differences in PI duration between the different intensities (Fig. 1C), although the number of fruits produced was affected (Fig. 1D). This suggests that light intensity has no effect on IM lifetime but does affect either the rate of flower opening or the rate at which floral primordia are initiated. Conversely, day length significantly affected PI duration but not the number of fruits produced (Fig. 1, E and F).

**Figure 2.**
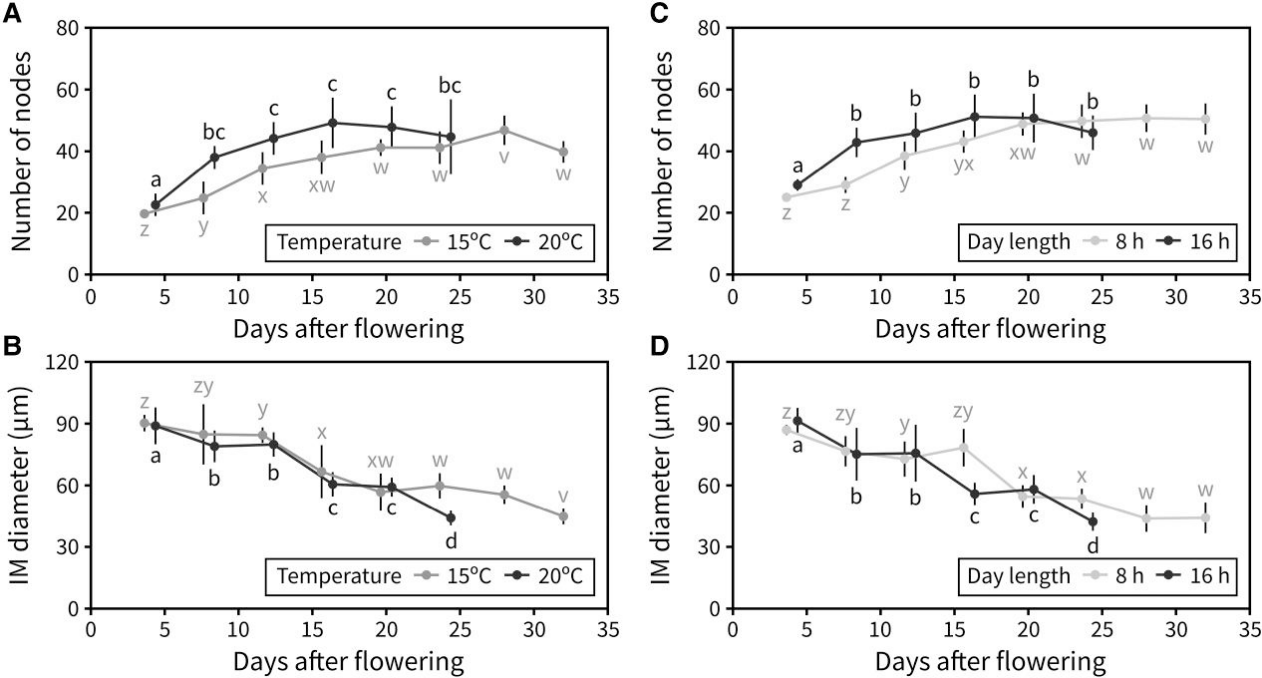
IM activity is controlled by day length and temperature. **A and B)** Effect of temperature on the activity of the IM. **A)** Average number of reproductive nodes (i.e. siliques, flowers, and floral primordia) produced along the PI at different time points through PI lifetime, in Col-0 plants grown at 15 °C or 20 °C (*n* = 4 to 7). **B)** Average diameter of the IM at different time points during PI lifetime in Col-0 plants grown at 15 °C or 20 °C (*n* = 4 to 7). **C and D)** Effect of day length on the activity of the IM. **C)** Average number of reproductive nodes (i.e. siliques, flowers, and floral primordia) produced along the PI at different time points through PI lifetime, in Col-0 plants grown under a 16- or 8-h photoperiod (*n* = 6 to 12). **D)** Average diameter of the IM at different time points during PI lifetime in Col-0 plants grown under a 16- or 8-h photoperiod (*n* = 6 to 12). Different letters indicate statistical differences between time points for a given environmental condition (ANOVA, Tukey HSD, *P* < 0.05). Error bars represent the standard deviation.

We focused on unraveling the effect of day length and ambient temperature on inflorescence duration and aimed to determine the developmental origin of the altered durations in response to different photo-thermal conditions. Specifically, we hypothesized that the shorter PI duration under higher temperatures and longer day lengths would be due to an earlier arrest of the IM. To test this, we tracked the number of reproductive nodes produced by the IM during flowering. Two separate experiments were designed for this, a first one with plants grown under either 8- or 16-h day lengths (22 °C) and a second one with plants grown under either 15 °C or 20 °C (16-h day length). In both cases, plants were kept under the flowering-inducing condition (16-h day length and 20 °C, respectively) during the vegetative phase, and the different treatments were applied after bolting. The IM was dissected out every 4 d to record the number of reproductive nodes initiated in a time-series manner, as previously described (Walker et al. 2023). For each experiment, the maximum number of nodes was the same in both treatments but was reached roughly ∼10 d earlier under a 16-h day length compared with an 8-h one (Fig. 2C), and over 10 d earlier under 20 °C compared with 15 °C (Fig. 2A). This suggested that an increase in photoperiod or ambient temperature accelerates the rate at which the IM initiates floral primordia, with a compensatory reduction in the duration of the PI. Previous work in the field has reported a gradual decrease in the size of the IM as flowering comes to an end (Wang et al. 2020), which was also found here under all conditions but started earlier under 16-h day length and 20 °C (Fig. 2, B and D; Supplemental Fig. S2). Taken together, these results pointed towards a model in which photoperiod and temperature, but not light intensity, regulate the developmental progression of the IM, including the timing of IM arrest.

### Photo-thermal exposure during flowering regulates arrest

**Figure 3.**
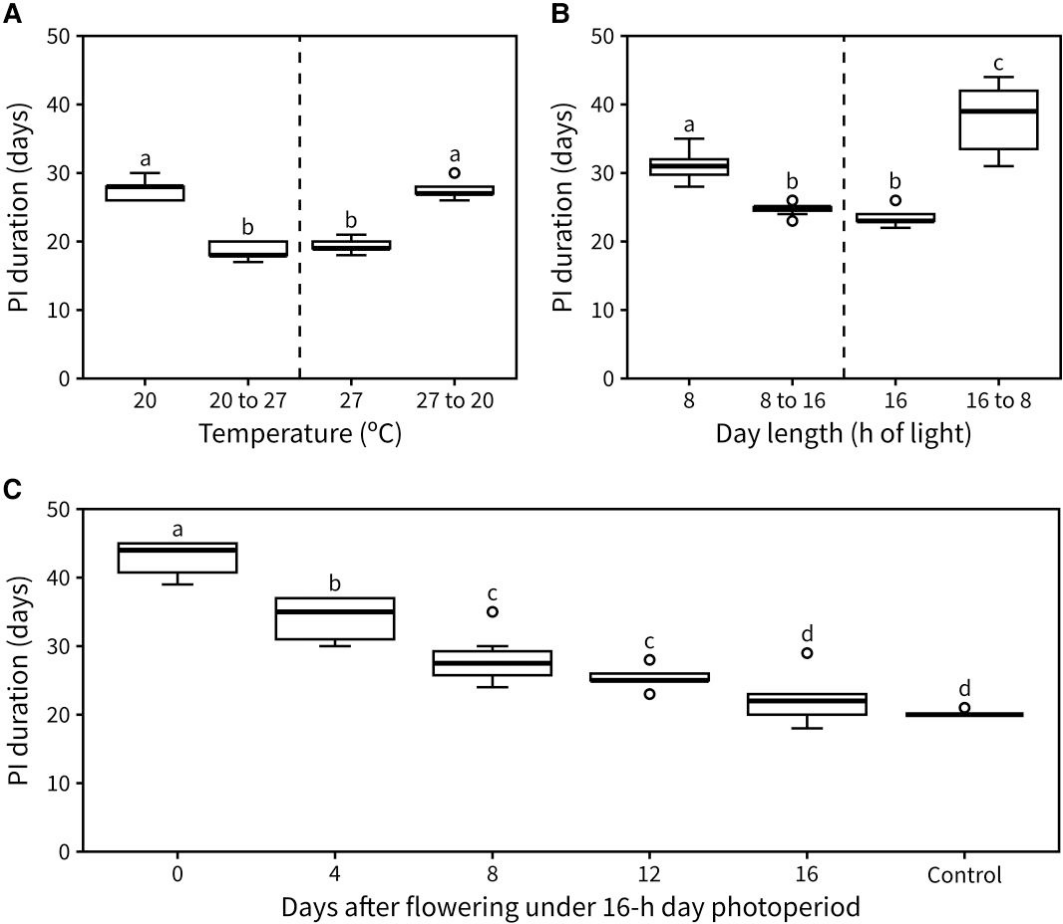
Photo-thermal exposure during flowering alone regulates arrest. **A)** Duration of the PI in plants exposed to either continuous 20 °C or 27 °C, or with a reciprocal transfer between those conditions upon visible bolting (*n* = 9 to 13). **B)** Duration of the PI in plants exposed to either continuous 8- or 16-h day length or with a reciprocal transfer between those conditions upon visible bolting (*n* = 7 to 12). **C)** Duration of the PI in plants transferred from a 16-h photoperiod to an 8-h one 0-, 4-, 12-, or 16-d post-flowering (*n* = 8 to 9). Control plants were never transferred to 8-h day length. Different letters indicate statistical differences (for A and B: ANOVA, Tukey test, *P* < 0.05, for C: Kruskal–Wallis rank sum test, pairwise Wilcoxon test with Benjamini and Hochberg correction, *P* < 0.05). Boxes indicate the interquartile range. The central line indicates the median, and whiskers show minimum and maximum values.

An interesting question raised by these results is whether the timing of inflorescence arrest is regulated by total light or thermal exposure during the vegetative and reproductive phases, or only the latter. To test this, we grew plants under either 20 °C or 27 °C and reciprocally transferred a subset of them to the other temperature upon bolting. Interestingly, a warmer temperature during flowering was sufficient to reduce PI duration regardless of the plant's previous experience. Conversely, a reduction in temperature led to a longer PI duration even in plants that had spent their vegetative phase at 27 °C (Fig. 3A). The same principle was then applied to test the effect of past day length on inflorescence arrest, with plants being grown under either a 16- or 8-h day length and reciprocally transferred between conditions upon bolting. Day length during flowering was the main determinant of PI duration (Fig. 3B), especially when comparing plants in continuous 16-h day length and plants transferred from an 8-h photoperiod.

Knowing that photo-thermal experience during flowering is the main driver of inflorescence arrest, we next questioned whether this responsiveness to light and temperature is present through the duration of the PI. To test this, we performed an experiment where plants were transferred from a 16-h day length to an 8-h one at a late stage into flowering (approximately 16 d after flowering). No statistical difference was found in the PI duration between transferred plants and the control that was maintained under a 16-h photoperiod (Supplemental Fig. S3). This suggested that sensitivity to day length is lost at some point into flowering. To further establish the approximate time at which plants become insensitive to photo-thermal cues, we set out a time-course experiment in which plants were grown under a 16-h day length until bolting. Next, subsets of plants were transferred to an 8-h day length after 0, 4, 8, 12, or 16 d of flowering, including a control group which stayed in a 16-h day length throughout its lifetime. We observed that the longer plants spent in the 8-h photoperiod, the longer the duration of the PI became, except for the 16-d transfer group which was statistically equivalent to plants that stayed under a 16-h day length (Fig. 3C). These data indicate that plants are sensitive to changes in day length throughout flowering, until the IM arrests (which occurs at around 16 d post-bolting in 16-h day lengths) (Fig. 2C).

**Figure 4.**
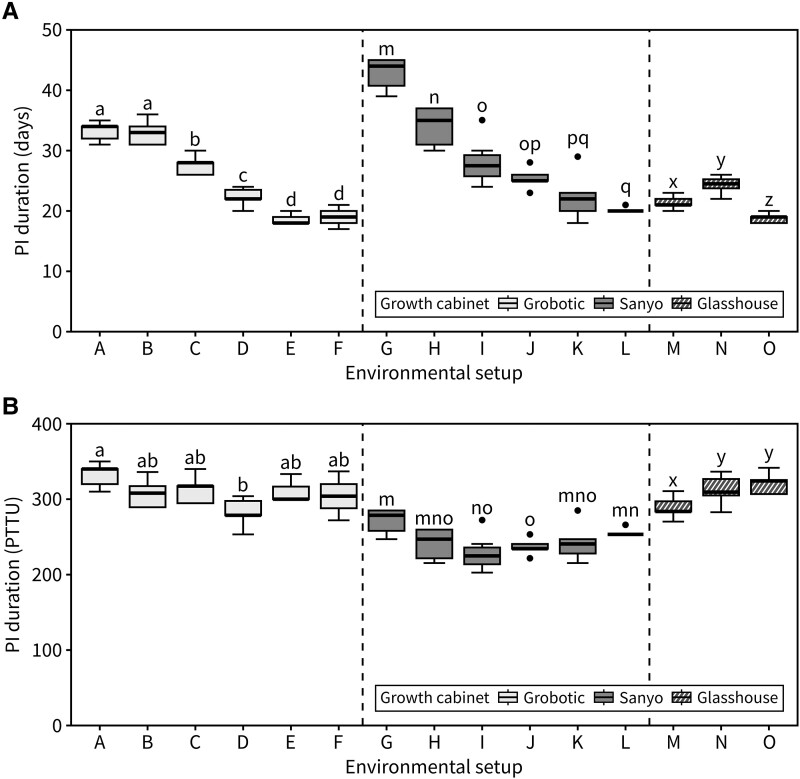
Inflorescence arrest occurs at a photo-thermal threshold. **A)** Duration of the PI in a range of environmental setups expressed in calendar time (days) (*n* = 7 to 13). Different letters indicate statistical differences between groups (for environmental setups A–D: ANOVA, Tukey's HSD test, *P* < 0.05, for E–O: Kruskal–Wallis rank sum test, pairwise Wilcoxon test with Benjamini and Hochberg correction, *P* < 0.05). **B)** Duration of the PI for the same data as **(A)** expressed in cumulative photo-thermal time units (PTTU, °C daylight days) (*n* = 7 to 13). Different letters indicate statistical differences between groups (for environmental setups A–L: Kruskal–Wallis rank sum test, pairwise Wilcoxon test with Benjamini and Hochberg correction, *P* < 0.05, for M–O: ANOVA, Tukey's HSD test, *P* < 0.05). Setups A–F are identical to Fig. 1A, G–L are identical to Fig. 3C, and M–O represent new data (see Supplemental Table S1). Boxes indicate the interquartile range. The central line indicates the median, and whiskers show minimum and maximum values.

Our results suggested that other factors being equal, day length and temperature conditions during flowering are sufficient to explain the timing of inflorescence arrest. The concept of photo-thermal time, which integrates daily temperature and light-hour exposure by plants, has often been used with success to predict the timing of floral transition. Following the same idea, we hypothesized that cumulative photo-thermal time during flowering would be sufficient to predict the timing of inflorescence arrest. To test this, we compiled data from a series of experiments manipulating both day length and temperature aiming to create a dataset of individual plants with a range of different photo-thermal exposures, some of which have already been discussed before (Fig. 4A: setups A–F are identical to Fig. 1A, G–L are identical to Fig. 3C, and setups M–O represent new data). In accordance with the data previously presented, PI duration was strongly affected by the environmental conditions during flowering. However, cumulative photo-thermal time at PI arrest was similar across different environmental setups (Fig. 4B). This was also true for experiments grown in glasshouses with less control over temperature, where the daily temperature was recorded and used to calculate the cumulative photo-thermal time (Fig. 4B, setups M–O). In an additional experiment, an artificial “warming” setup was designed where both temperature and photoperiod increased over time. Despite the absolute PI duration (in days) being significantly shorter in the “warming” regime, the final number of experienced photo-thermal time units was equivalent to control plants that did not experience warming (Supplemental Fig. S4), further supporting the idea that reaching a certain photo-thermal threshold is required for inflorescence arrest.

### The photoperiod and ambient temperature flowering pathways control photo-thermal inflorescence arrest

**Figure 5.**
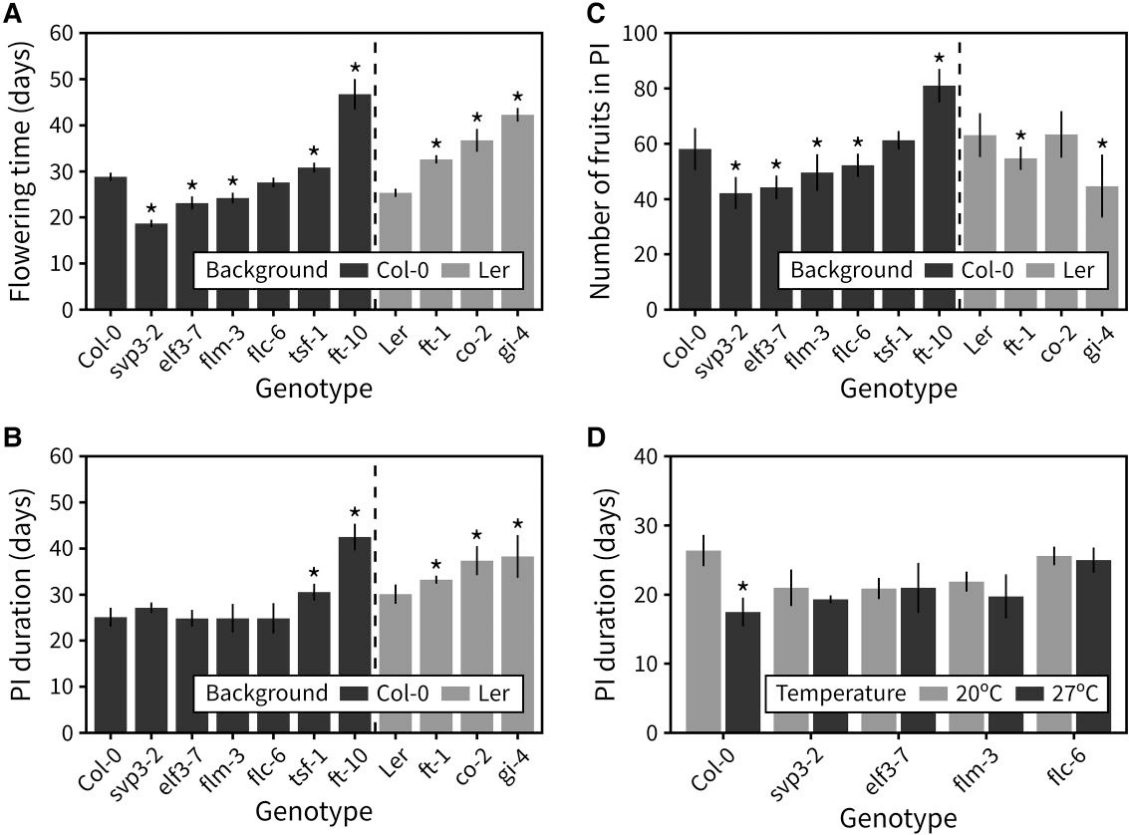
The photoperiod and ambient temperature flowering pathways control photo-thermal arrest. **A)** Flowering time in an array of mutants deficient in the ambient temperature flowering pathway, the photoperiod flowering pathway, or both (*n* = 4 to 8). **B)** Duration of the PI in the same array of mutants. **C)** The number of fruits produced in the PI in the same array of mutants. In (**A–C)**, asterisks indicate statistical differences between a mutant and the appropriate wild type (Kruskal–Wallis rank sum test, pairwise Wilcoxon test with Benjamini and Hochberg correction, *P* < 0.05). **D)** Duration of the PI in an array of ambient temperature pathway mutants grown at 20 °C or 27 °C (*n* = 4 to 6). In (**D)**, asterisks indicate statistical differences within each genotype and between the 2 temperatures (Student's *t*-test, *P* < 0.05). Error bars represent the standard deviation.

We next aimed to identify the underlying genetic mechanisms that regulate inflorescence responses to photo-thermal time. To do so, we screened a range of Arabidopsis mutants deficient in genes involved in the ambient temperature flowering pathway *FLOWERING LOCUS M* (*FLM*; *flm-3*), *FLOWERING LOCUS C* (*FLC; flc-6*), and *SHORT VEGETATIVE PHASE* (*SVP*; *svp-32*), the photoperiod pathway *GIGANTEA* (*GI*; *gi-4*) and *CONSTANS* (*CO*; *co-2*), or *EARLY FLOWERING 3* (*ELF3*; *elf3-7*), *FLOWERING LOCUS T* (*FT*; *ft-10*), and *TWIN SISTER OF FT* (*TSF*; *tsf-1*) for differences in PI duration. All the mutants, together with the wild-type backgrounds were grown at standard conditions (22 °C, 16-h day length, 100 *μ*mol s^−1^ m^−2^). Although all mutants were affected in the length of the vegetative phase (Fig. 5A), only a few showed differences in PI duration compared with wild type (Fig. 5B). All mutants deficient in components of the photoperiod pathway showed a delayed inflorescence arrest. Of these, *ft-10*, which lacks the key floral integrator *FT*, showed the longest PI duration in the Col-0 background and produced more fruits (Fig. 5C, Supplemental Fig. S5). Inspection of an independent allele in Ler (*ft-1*) ruled out a background-specific effect. Interestingly, early flowering mutants *svp3-2* and *elf3-7* had the same PI duration as wild type, demonstrating that vegetative and reproductive duration are not necessarily coupled. Accordingly, the 2 wild-type accessions included here (Col-0 and Ler) had the same lifespan but opposite patterns in the time allocation to developmental phases, with Col-0 having a longer vegetative period and Ler having a longer reproductive period.

That none of the mutants deficient in the ambient temperature pathway showed differences in PI duration compared with wild type was surprising, especially considering the clear role of temperature in controlling inflorescence duration (Fig. 1A). We thus hypothesized that the ambient temperature pathway may be necessary for the differences in PI duration seen when growing wild-type plants at different temperatures. To test this, we grew the subset of mutants deficient in this pathway under 2 different temperatures, 20 °C and 27 °C (16-h day length, 100 *μ*mol s^−1^ m^−2^), and recorded their inflorescence duration. Other than the wild type, none of the genotypes showed statistically significant differences in duration between the 2 temperatures (Fig. 5D). This demonstrates that although mutants in the ambient temperature pathway do not show differences relative to wild type at 22 °C (Fig. 5B), the ambient temperature pathway is required for PI duration response to temperature during flowering.

### *FT* expression increases with photo-thermal time

**Figure 6.**
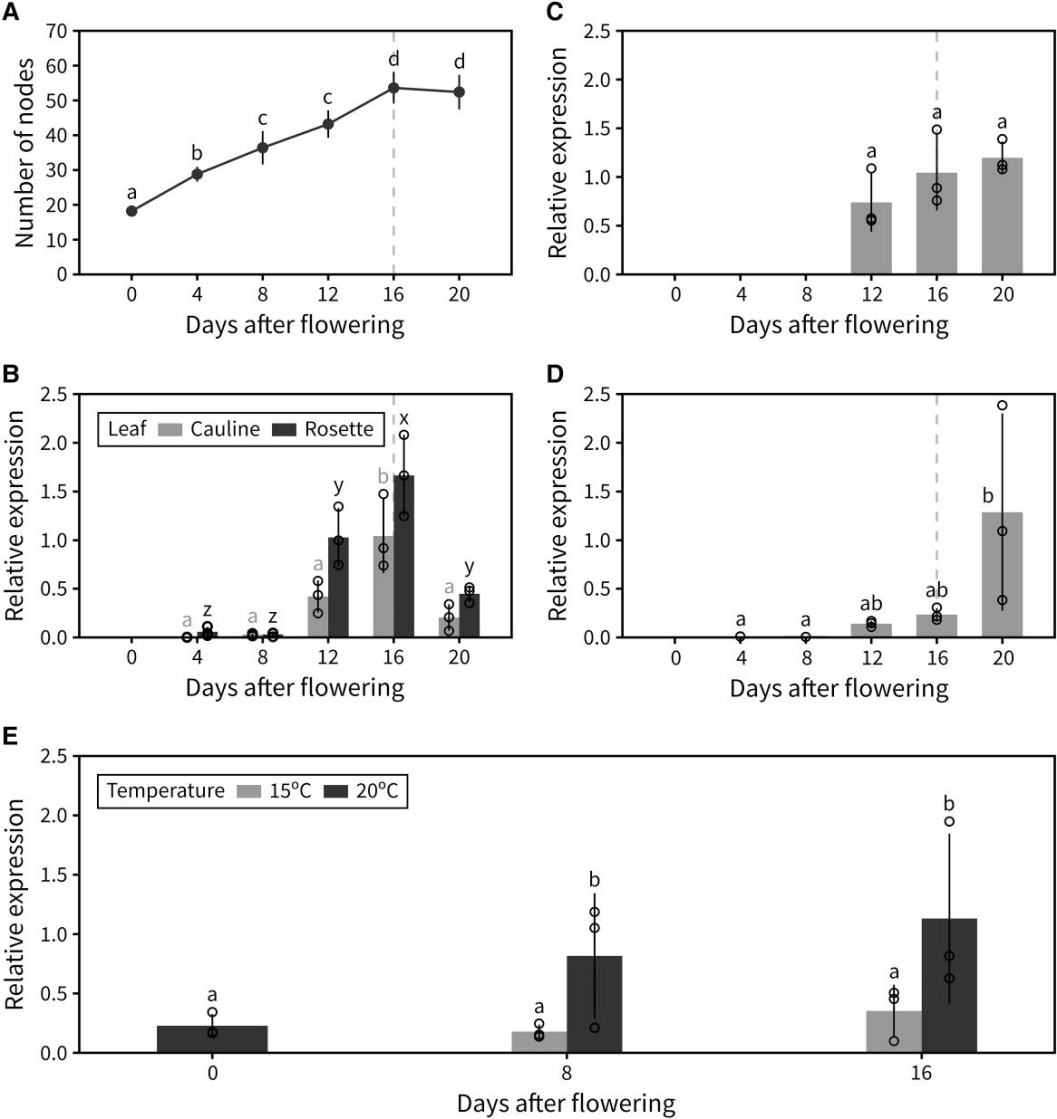
Global *FT* expression increases with photo-thermal time. **A)** Cumulative number of reproductive nodes (i.e. siliques, flowers, and floral primordia) produced at different points during PI lifetime in the plant population used for RT-qPCR (*n* = 5). **B–D)** Relative transcript level of *FT*, assessed by RT-qPCR, in leaves (both rosette and cauline) **(B)**, siliques **(C)**, and the IM **(D)** at different points during PI lifetime. Error bars indicate the standard deviation from the average of biological replicates (*n* = 3). Transcript levels were normalized to *UBC9* and then to the maximum *FT* level. Approximate timing of IM arrest is indicated by a dashed vertical line. **E)** Relative transcript level of *FT*, assessed by RT-qPCR, in rosette leaves at different time points after flowering in plants grown at 15 °C or 20 °C. Error bars indicate the standard deviation from the average of biological replicates (*n* = 3). Transcript levels were normalized to *ACT1* and then to the maximum *FT* level. In **(A–E)**, different letters indicate statistical differences (ANOVA, Tukey's HSD test, *P* < 0.05). Error bars represent the standard deviation.

Based on the phenotype of *ft-10* and *ft-1* mutants, we hypothesized that photo-thermal regulation of *FT* at the transcriptional level could underlie the regulation of IM arrest. To explore this idea, we performed a detailed analysis of *FT* expression over the course of flowering in different plant tissues. In addition to harvesting tissue for reverse transcription-quantitative PCR (RT-qPCR), we performed time-course dissections of plants in the same experiment to determine the approximate time of IM arrest in this experiment. Under our growing conditions (22 °C, 16-h day length), IM arrest took place ∼16 d post-bolting (Fig. 6A). *FT* transcript levels in the leaves are known to increase over time during the vegetative phase and to reach an expression peak prior to floral transition. However, expression dynamics of *FT* at later time points have been largely overlooked and are only beginning to be understood (Liu et al. 2014; Müller-Xing et al. 2014). Previous work has suggested that *FT* expression after floral transition helps stabilize the IM, allowing reproductive development to continue regardless of the environment. Our data show that *FT* expression in both rosette and cauline leaves shows a previously uncharacterized peak of expression approximately 2 wk after floral transition (Fig. 6B). This peak of *FT* expression in the leaves occurred around the same time as IM arrest, suggesting that *FT* expressed in the leaves could play a role in IM arrest. Interestingly, the same applied to *CO*, a known upstream regulator of *FT*, whose mRNA levels also peaked ∼16 d post-bolting in the rosette leaves (Supplemental Fig. S6). In other plant tissues, *FT* expression followed a somewhat similar pattern, with slightly higher transcript levels in siliques later in flowering (Fig. 6C). We also observed a clear transcriptional activation of *FT* in the IM itself, where *FT* is not normally expressed, after arrest (Fig. 6D). As we have previously shown, IM arrest seems to occur after a certain photo-thermal exposure has been experienced by the plant, so we hypothesized that transcriptional upregulation of *FT* might be the underlying internal “counter” of experienced photo-thermal units during flowering. To test this, we grew plants at either 20 °C or 15 °C and measured *FT* transcript levels in the leaves at different time points after flowering. *FT* transcription is upregulated over time during flowering under both conditions. However, the transcriptional activation of *FT* occurs significantly more slowly at 15 °C, suggesting that *FT* transcript builds up specifically in response to photo-thermal time and not simply absolute plant age or time since flowering (Fig. 6E).

We hypothesized that the transcriptional dynamics observed for *FT* would translate into an increase in FT protein in the IM over the course of flowering. We tested this by examining the previously described transgenic *FTpro:FT-GFP* line by confocal microscopy. However, we were unable to detect a GFP signal in IMs and note that the GFP images from the original publication are not quantified, nor do they clearly show GFP in the IM itself. Thus, we deemed this tool unusable to address this question, though we do not doubt that FT accumulates in shoot meristems.

### *FT* expression mediates photo-thermal timing of IM arrest

**Figure 7.**
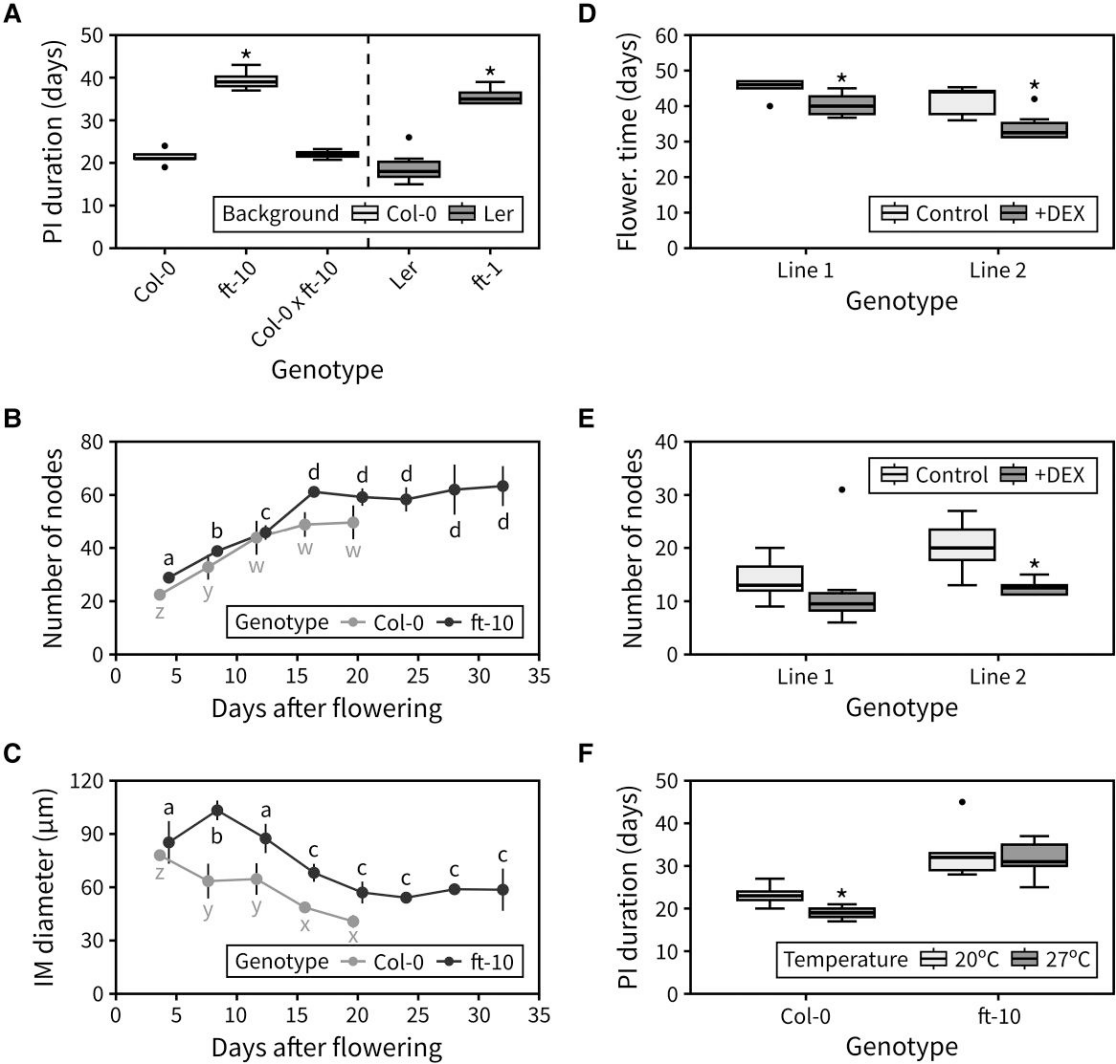
*FT* mediates the arrest of the IM. **A)** Duration of the PI in the *FT* mutants, and the heterozygous Col-0*×ft-10* (*n* = 3 to 8). In **(A)**, asterisks indicate statistical differences between a mutant and the appropriate wild type (Kruskal–Wallis rank sum test, pairwise Wilcoxon test with Benjamini and Hochberg correction, *P* < 0.05). **B)** Cumulative number of reproductive nodes (siliques, flowers, and floral primordia) produced at different points during PI lifetime in Col-0 and *ft-10* mutants (*n* = 3 to 6). **C)** Average diameter of the IM at different time points during PI lifetime in Col-0 and *ft-10* mutants (*n* = 3 to 6). In **(B–C)**, different letters indicate statistical differences between time points for a given genotype (ANOVA, Tukey HSD, *P* < 0.05). **D and E)** Flowering time **(D)** and final number of reproductive nodes **(E)** in 2 independent *35S:FT-GR* lines grown on agar-solidified media with or without 10 *μ*M DEX (*n* = 3 to 12). In **(D and E**), asterisks indicate statistical differences within each line (2-sample Wilcoxon test, *P* < 0.05). **F)** Duration of the PI in Col-0 and *ft-10* mutants exposed to different ambient temperatures during flowering (*n* = 12). In **(F)**, asterisks indicate statistical differences between temperatures for each genotype (Student's *t*-test, *P* < 0.05). Boxes indicate the interquartile range. The central line indicates the median, and whiskers show minimum and maximum values. Error bars represent the standard deviation.

Compared with the difference in PI duration between *ft-10* and Col-0 (∼20 d), the duration of the *ft-1* mutant was only ∼5 d longer than the Ler wild type, albeit significantly different. However, in a second run of the experiment, *ft-1* showed a much longer duration, and a difference to the wild type comparable to that of *ft-10* to Col-0 (Fig. 7A). Furthermore, analysis of heterozygous Col-0*×ft-10* plants revealed that a single functional copy of *FT* is sufficient for timely inflorescence arrest (Fig. 7A). We next questioned whether *FT* controls the arrest of the IM specifically. To test this, we performed time-course dissections of the IM in the *ft-10* mutant and recorded the number of cumulative reproductive nodes during flowering, as well as the average diameter of the IM. This revealed that inflorescence arrest is delayed in *ft-10* because the lifetime of the IM is extended by ∼5 d relative to wild type. The rate at which the IM initiates floral primordia is the same in Col-0 and *ft-10* and, because of the prolonged span of the IM, the final number of fruits on the PI is also, therefore, significantly increased in *ft-10* mutants (Fig. 7B). Interestingly, despite showing similar IM sizes shortly after floral transition, the IM of *ft-10* mutants increases in diameter during flowering and is overall bigger than that of the wild type during flowering (Fig. 7C). These results demonstrate that functional *FT* is required for timely IM arrest.

Overall, the global expression of *FT* seems to peak at IM arrest (Fig. 6), suggesting that a certain threshold level of *FT* expression is necessary for timely IM arrest. To test whether FT expression is sufficient to promote inflorescence arrest, we transformed a *35S:FT-GR* construct into the Col-0 background. This construct over-expresses *FT* from the constitutive *35S* promoter, but because of the fusion of the FT protein to the rat glucocorticoid receptor (GR), FT is excluded from the nucleus (and therefore inactive) unless a steroid hormone such as dexamethasone (DEX) is added. We thus used the resultant transgenic lines to test whether inflorescence arrest could be accelerated by DEX-inducing *FT* over-expression. *35S:FT-GR* promoted very early floral transition (and highly pleiotropic effects) in most lines when grown on agar-solidified media containing DEX. However, we selected the 2 lines with the weakest phenotype for further analysis (Fig. 7D). These lines demonstrated that the induction of *FT* accelerates floral transition and reduces PI duration leading to plants having a lower number of fruits in the PI (Fig. 7E, Supplemental Fig. S7).

Finally, to test whether FT is necessary for photo-thermal promotion of inflorescence arrest, we assessed whether ft-10 mutants are able to respond to changes in photo-thermal exposure. While wild-type plants showed a characteristic reduction in PI duration at warmer temperatures, we observed that *ft-10* mutants had the same PI duration under both warmer and colder temperatures (Fig. 7F). Collectively, our results, therefore, support the idea that photo-thermally regulated *FT* expression drives both the floral transition and the arrest of the IM at the end of flowering.

## Discussion

The data presented here show that the flowering phase is highly sensitive to photo-thermal cues and challenges the idea that the molecular machinery that underlies the perception of such cues is merely tasked with the stabilization of flowering (Liu et al. 2014). Rather, our results suggest the existence of an internal timekeeper which tracks long-term accumulation of photo-thermal units to properly time arrest at both the IM and inflorescence levels. Although such a mechanism has been described before in the context of floral transition (Masle et al. 1989; Song et al. 2015), our work demonstrated that photo-thermal timekeeping continues to take place after the onset of flowering. Our working model suggests that reaching a photo-thermal threshold is sufficient to trigger IM arrest. However, this raises the question as to whether the “path” that leads to this threshold matters, and specifically, how fluctuating light and temperature conditions affect the timing of IM arrest. As such, future studies would benefit from the use of a more complex gradient of conditions or field trials, whose outcome is typically harder to predict when compared with constant lab conditions (Kinmonth-Schultz et al. 2019). The need to further characterize end-of-flowering responses to photo-thermal cues is evident, particularly when considering the current global warming scenario, since early end-of-flowering is triggered by warmer temperatures and might act as a structural limit on crop yields. Our work is an important step towards predicting how the duration of flowering in crop species will be affected in such a warming climate.

The role of *FT* in the timing of floral transition has been extensively described (Turck et al. 2008; Srikanth and Schmid 2011; Song et al. 2015). Specifically, reaching a threshold of *FT* expression is sufficient to trigger transition into flowering, at least under most conditions (Kinmonth-Schultz et al. 2019). Since FT acts as a mobile leaf-to-meristem signal (Corbesier et al. 2007), it has been characterized as “florigen”, the mobile flowering-promotive signal identified in Chailakhyan's seminal grafting experiments. While typically described as “part of florigen”, it has also become common to co-equate FT and florigen (Corbesier et al. 2007; Turck et al. 2008); either way, the primary conception of FT function is that it is a specific activator of flowering. However, a growing body of publications from the last few years (e.g. Kinoshita et al. 2011; Navarro et al. 2011; Chen et al. 2014), to which our work adds, challenges this FT-as-florigen conceptualization. Our data suggest that photo-thermally controlled regulation of *FT* at the transcriptional level drives the arrest of the reproductive program in Arabidopsis. Based on the data presented here, we propose that *FT* expression is the output of a generalized “photo-thermal stopwatch” that measures cumulative photo-thermal time during the whole plant's lifetime. We believe that FT is, therefore, not simply an instruction to flower, but a systemically distributed form of information that allows different developmental processes to be correctly timed with respect to photo-thermal exposure. Previous research on the end-of-flowering has focused on the idea that a signal produced in the fruits drives reproductive arrest in Arabidopsis (Hensel et al. 1994), and the implication of auxin in this mechanism has been shown at the level of floral arrest (Ware et al. 2020; Walker et al. 2023). Given that *FT* is expressed in siliques throughout flowering, it is tempting to hypothesize that mobile FT protein may also form a component of this seed-derived signal. This provides a clear avenue for future investigation. We propose that the floral transition and IM arrest developmental processes both measure FT concentration to modulate their timing (albeit with very different sensitivities), but that neither process is dependent on FT to occur, consistent with the phenotype of *ft* mutants. The question remains as to what role the circadian clock exerts on this process, especially given the interesting phenotype of *gi* and *co* mutants, and the transcriptional dynamics of these genes throughout flowering. Future work would benefit from an effort to better understand the regulation that occurs upstream of *FT*. At any rate, there is clear evidence that FT expression is downregulated at the end-of-flowering (Satake et al. 2013; Miryeganeh et al. 2018), which suggests that perennial plants may “reset” their photo-thermal stopwatch with each reproductive cycle, allowing them to correctly time each subsequent phase, irrespective of past environmental conditions. Our model may also provide an explanation for the proliferation of *FT* paralogues in monocots (Bennett and Dixon 2021). It may be that these duplicated *FT*s are the outputs of different forms of time (or environmental) exposure in monocots, while still being able to influence the same range of developmental processes through interaction with FD homologs. Overall, our data contribute to an emerging picture of FT proteins as broad, systemic regulators of development and provide a platform for further analysis of these fascinating proteins.

## Materials and methods

### Plant material and growth conditions

Arabidopsis (*A. thaliana*) plants in the Col-0 background were used for all plant growth experiments, unless otherwise specified. Mutants used in this work have been previously described: *svp-32* (Lee et al. 2007), *elf3-7* (Hicks et al. 2001), *flc-6* (Schönrock et al. 2006), *gi-4* (Koornneef et al. 1991), *co-2* (Koornneef et al. 1991), *flm-3* (Posé et al. 2013), *tsf-1* (Yamaguchi et al. 2005), *ft-10* (Yoo et al. 2005), and *ft-1* (Koornneef et al. 1991). All were in the Col-0 background except *gi-4*, *co-2*, and *ft-1* (Ler background). Seeds were germinated on 100 ml pots containing a 2:1 (v/v) mixture of Petersfield No.2 Supreme Compost and perlite, where plants were kept until senescence. Most experiments were performed in plant growth chambers (Grobotic Systems) and controlled environment rooms (Sanyo), with light provided by fluorescent tubes. Some experiments were performed in glasshouses where the day length was extended to 16 h light. The temperature in the glasshouse was registered daily with a thermometer. All standard growth conditions used throughout the study are listed in Table 1. Unless otherwise stated, the light intensity was 100 *μ*mol s^−1^ m^−2^. The duration of the light/dark cycles is 24 h and the day length is reported through the text as the hours of light per 24 h cycle. Additional experiments are specified in Supplemental Table S1.

**Table 1.** Standard growth conditions used throughout the study

Condition	Light intensity (*μ*mol m^−2^ s^−1^)	Day length (h of light)	Temperature (°C)	
			Daytime	Nighttime
27 °C	100	16	27	25
25 °C	100	16	25	25
22 °C	100	16	22	20
20 °C	100	16	20	20
17 °C	100	16	17	15
15 °C	100	16	15	15
50 *μ*mol m^−2^ s^−1^	50	16	22	22
150 *μ*mol m^−2^ s^−1^	150	16	22	22
100 *μ*mol m^−2^ s^−1^	100	16	22	22
16 h of light	120	16	20	20
8 h of light	120	8	20	20

### *35S:FT-G* *R* generation

Transgenic *35S:FT-GR* lines were generated by cloning the full coding sequence (CDS) of *FLOWERING LOCUS T* (*FT*) (AT1G65480) from *A. thaliana* downstream of the CaMV 35S promoter and fused to a GR tag. Gateway Cloning technology (Thermo Fisher Scientific) was used to clone the *35S* promoter and the CDS separately and to further assemble both into a modified pFP101 vector already containing the GR tag. *Agrobacterium tumefaciens* (GV1101) was used to transform *A. thaliana* plants using the floral dip method. Independent T2 lines carrying the transgene insertion were selected and used for the in vitro experiments. All primers used for the cloning of *35S:FT-GR* are listed in Supplemental Table S2.

### Recording of timing data

After germination, plants were checked twice a week for signs of visible bolting. Bolting was recorded as the first date on which a cluster of floral primordia could be observed in the center of the rosette. After flowering started, the number of flowers in the PI was counted 3 times a week. Inflorescence arrest was recorded as the last date on which a new flower was opened in the PI. Inflorescence duration was then calculated as the difference in days between bolting and inflorescence arrest. Timing data were also scaled in photo-thermal units as previously described (Masle et al. 1989; Brachi et al. 2010). Formula for the calculation of photo-thermal time (PTT) is given as follows:


(1) $$PTT=\overset{n}{\underset{i=1}{\sum }}\;l_{i}(T_{i}-T_{b})=\overset{n}{\underset{i=1}{\sum }}\;l_{i}(T_{i}-3).\;$$

Photo-thermal time during *n* days is calculated as the sum for those *n* days of the proportion of hours of light per day *l_i_* multiplied by the excess of daytime average temperature *T_i_* above a base temperature *T_b_* (for *A. thaliana*, $T_{b}=3$).

### Meristem dissections

For experiments involving a characterization of the IM, the PI was sampled from plants at specific time points as indicated in the text and figures. The number of siliques and opened flowers was recorded for each inflorescence. Next, unopened floral primordia were dissected out of the apex under a digital microscope (Keyence), leaving the reproductive meristem exposed. The number of floral primordia was recorded in the process, and the exposed meristem was imaged. Image processing software (ImageJ) was used to measure the meristem diameter from the photographs.

### Reverse transcription-quantitative PCR

For RT-qPCR experiments, different plant tissues were harvested from Col-0 wild-type plants at different time points into flowering as specified in the text and figures. Mature rosette and cauline leaves of similar developmental ages were collected. Green siliques were harvested from the uppermost part of the inflorescence. Reproductive meristems were dissected out of the PI by removing all floral primordia under a dissecting scope (Olympus). Three biological replicates were sampled. RNeasy Plant Mini Kit (Qiagen) was used to extract the RNA, which was subsequently treated with TURBO DNA-free kit (Invitrogen) and used as a template to synthesize cDNA with a Transcriptor First Strand cDNA synthesis kit (Roche). Quantitative PCR was performed using PowerUp SYBR Green Master Mix (AppliedBiosystems) in a CFX Connect Real-time System (BioRad). Transcript fold change was obtained through normalization to the housekeeping gene: *UBIQUITIN CONJUGATING ENZYME 9* (*UBC9*) or *ACTIN 1* (*ACT1*). All primer sequences used for RT-qPCR are detailed in Supplemental Table S3.

### In vitro experiments

Work involving phenotyping the *35S:FT-GR* lines was carried out in vitro. Seeds were surface-sterilized with incubation in 70% (v/v) ethanol for 5 min, followed by a second incubation in 7% (v/v) bleach for 15 min, and lastly, a total of 5 sequential washes with sterile water. Four surface-sterilized seeds were sown per glass jar, each containing 200 mL of 0.8% (w/v) agar-solidified *A. thaliana* salts (ATS) media adjusted at pH 5.6 with 1% (w/v) sucrose and 10 *μ*M DEX (Supplemental Fig. S8). Jars were then placed in the dark at 4 °C for seed stratification for 2 d, after which they were transferred to a constant temperature room at 20 °C and 16 h day length with light supplied by fluorescent tubes (at ∼120 *μ*mol m^−2^ s^−1^). The timing of bolting was recorded, and plants were kept until no more opened flowers were visible, at which point the plants were harvested and the number of reproductive nodes in the PI (siliques and undeveloped floral primordia) was counted.

### Experimental design and statistical analysis

Sample sizes for each experiment are specified in the figure legends. For plant growth experiments, each sample is a distinct plant. For RT-qPCR experiments, each biological replicate consisted of 1 leaf, 1 silique, or a pool of 30 meristems. Data were analyzed with the statistical software R. For sample comparisons, data were tested for normality to determine the most appropriate statistical test. The specific test used in each case is indicated in the figure legends.

### Accession numbers

Sequence data from this article can be found in the GenBank/EMBL data libraries under accession numbers: FT (AT1G65480), FLM (AT1G77080), FLC (AT5G10140), SVP (AT2G22540), GI (AT1G22770), CO (AT5G15840), ELF3 (AT2G25930), TSF (AT4G20370), UBC9 (AT4G27960), ACT1 (AT2G37620).

## Supplementary Material

kiad163_Supplementary_Data

## Data Availability

All figures in this manuscript are associated with raw data, which will be made available upon request.

## Supplementary data

The following materials are available in the online version of this article.

**Supplemental Figure S1**. Temperature regulates the production rate of reproductive nodes.

**Supplemental Figure S2**. Inflorescence meristem size during flowering under 2 different day lengths.

**Supplemental Figure S3**. Effect of a late photoperiod transfer on the duration of the PI.

**Supplemental Figure S4**. Effect of an artificially imposed “warming” regime on inflorescence duration.

**Supplemental Figure S5**. Phenotype of *ft-10* mutants after end-of-flowering.

**Supplemental Figure S6**. Expression of *GI* and *CO* during flowering.

**Supplemental Figure S7**. Phenotype of the *35S:FT-GR* lines.

**Supplemental Figure S8**. Photographs illustrating the in vitro setup for the characterization of the *35S:FT-GR* lines.

**Supplemental Table S1**. Description of environmental setups used for Fig. 4.

**Supplemental Table S2**. Primers used for cloning of *35S:FT-GR*.

**Supplemental Table S3**. Primers used for RT-qPCR.
