# Supplementary material for: FLOWERING LOCUS T mediates photo-thermal timing of inflorescence meristem arrest in Arabidopsis thaliana
Source: Plant Physiol. 2023 Mar 21;192(3):2276–89. doi: 10.1093/plphys/kiad163 (PMC10315265; doi:10.1093/plphys/kiad163)
Supplement: kiad163_Supplementary_Data [file kiad163_supplementary_data.pdf]

SUPPLEMENTARY DATA

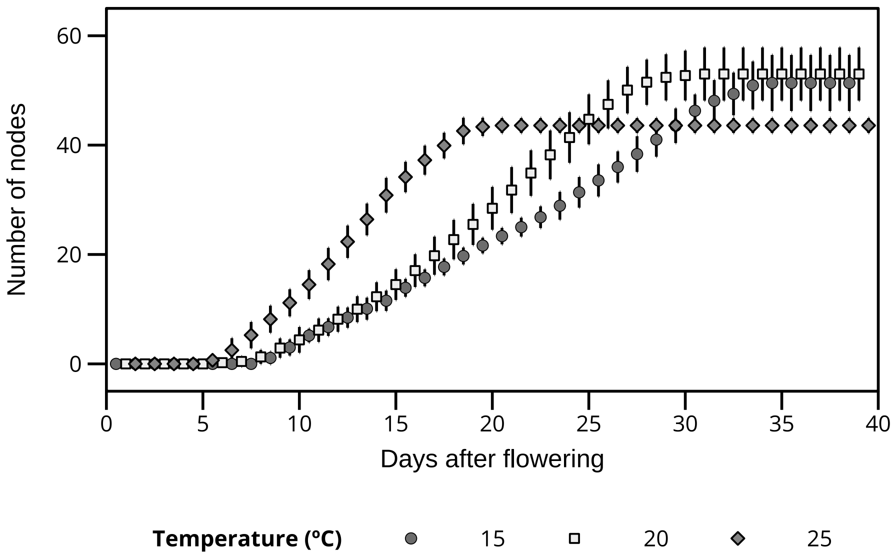

Supplementary Figure S1 | Temperature regulates the production rate of reproductive nodes.

Cumulative number of reproductive nodes (siliques and flowers) produced at different points during PI lifetime in Col-0 grown at 15ºC, 20ºC or 25ºC (n=6). Error bars represent the standard deviation (SD).

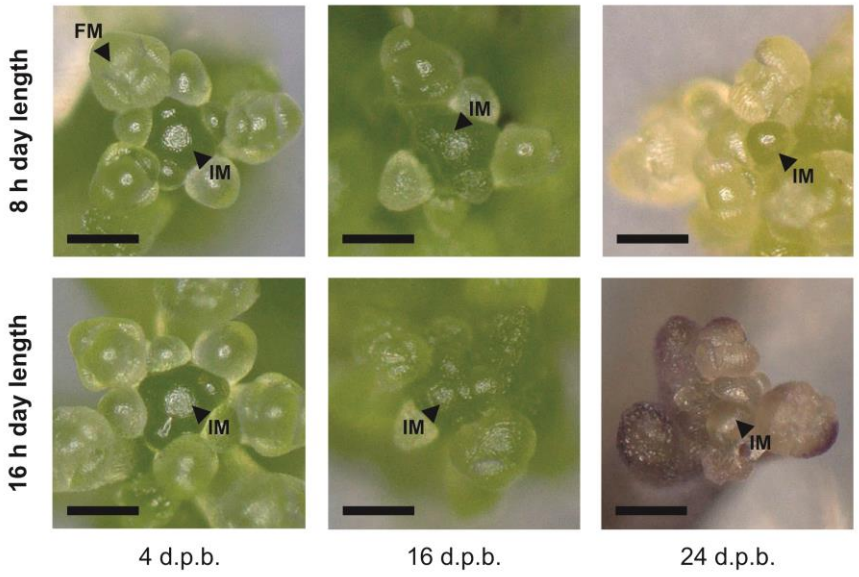

Supplementary Figure S2 | Inflorescence meristem size during flowering under two different day lengths.

Light micrographs showing the developmental progression of the IM during PI lifetime in Col-0 plants grown under a 16- or 8-hour photoperiod. Both groups transitioned into flowering under a 16-hour day length ~25 days after sowing, and a subset of plants was transferred to an 8-hour day length after bolting. The decline in meristem size, as well as its visible senescence, occurs later under an 8-hour day length. The scale bar represents 100 μm. FM: Floral meristem. IM: Inflorescence meristem. D.P.B.: Days post bolting.

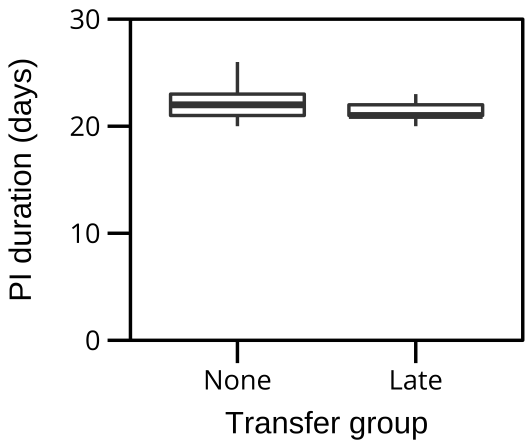

Supplementary Figure S3 | Effect of a late photoperiod transfer on the duration of the PI.

Duration of the primary inflorescence (PI) in plants that were grown under a 16-hour photoperiod until inflorescence arrest without transfer (‘none’) and those transferred to an 8-hour day length after ~16 days of flowering (‘late’) (n=9-12). No statistical differences were found (Student’s t test, $p<0.05$). Boxes indicate the interquartile range. The central line indicates the median, whiskers show minimum and maximum values.

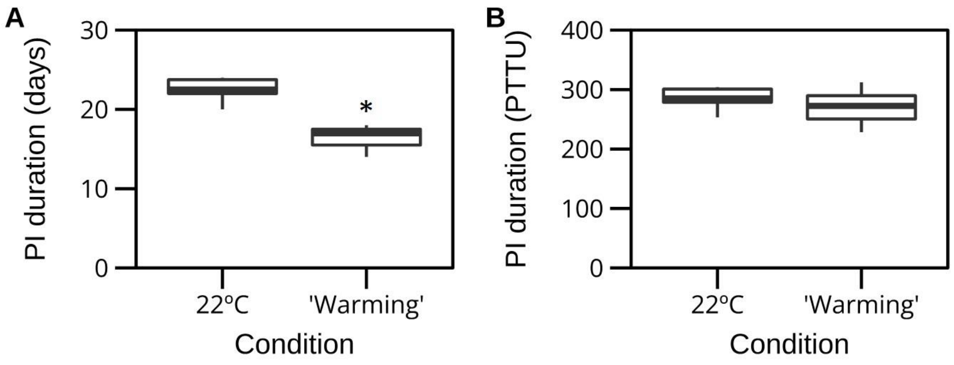

Supplementary Figure S4 | Effect of an artificially-imposed ‘warming’ regime on inflorescence duration.

(A-B) Duration of the primary inflorescence (PI) in plants that were grown at continuous 22ºC or a complex ‘warming’ environmental regime. Under the ‘warming’ regime, temperature was increased by 1ºC and day length by 1 hour every four days (n=6). **(A)** Duration of the PI in calendar time (days). **(B)** Duration of the PI for the same data as (A) expressed in cumulative photo-thermal units (PTTU, ºC daylight days). Asterisks indicate statistical differences (Student’s t test, $p<0.05$). Boxes indicate the interquartile range. The central line indicates the median, whiskers show minimum and maximum values.

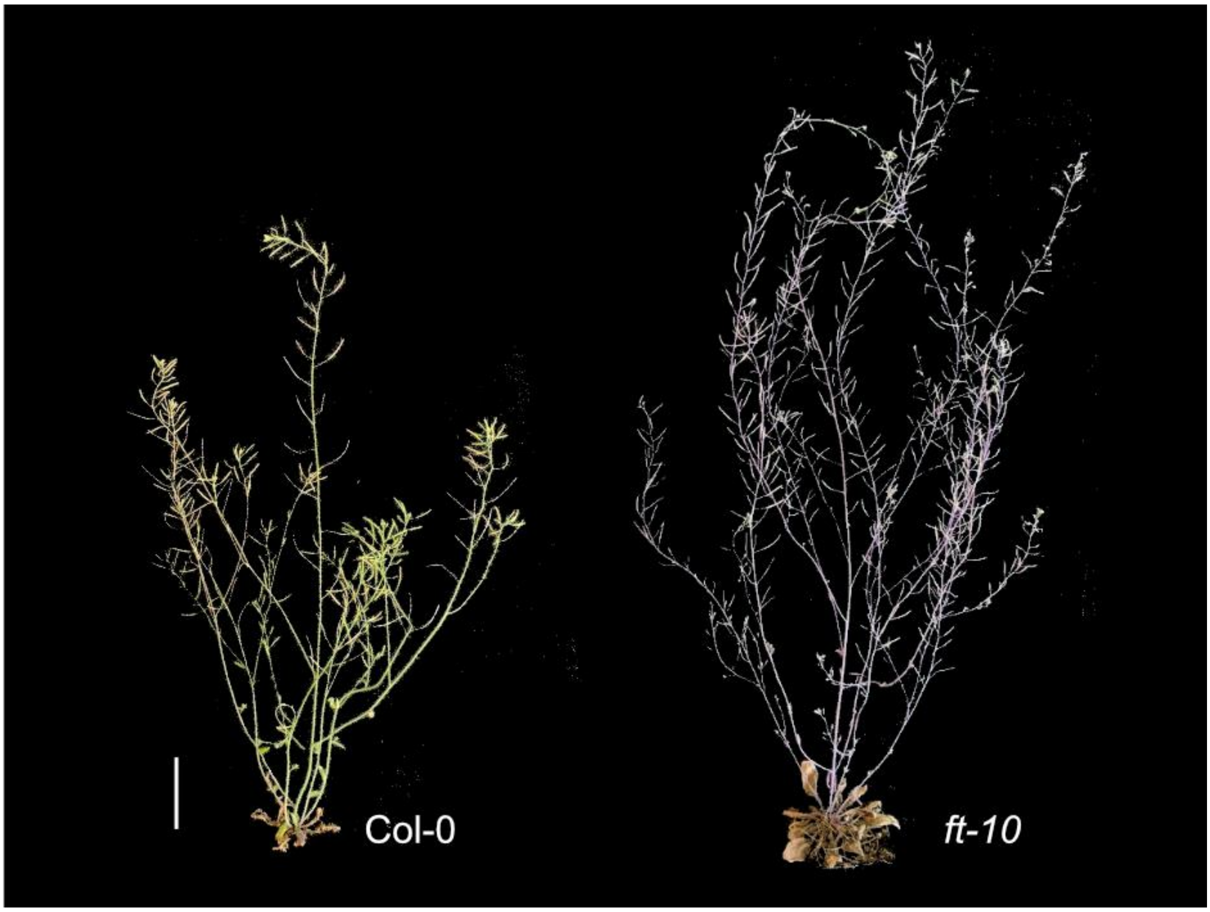

Supplementary Figure S5 | Phenotype of *ft-10* mutants after end-of-flowering.

Photographs of Col-0 (left) and *ft-10* (right) after end-of-flowering. The scale bar represents 5 cm.

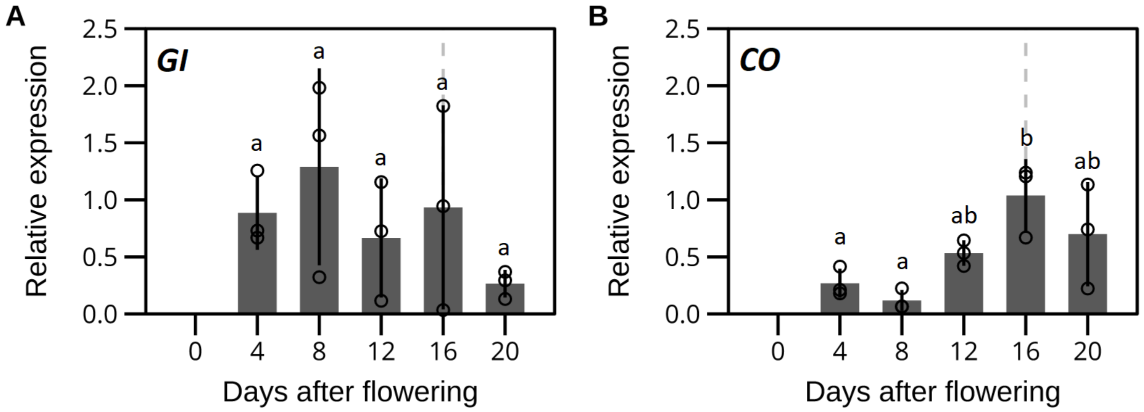

Supplementary Figure S6 | Expression of *GI* and *CO* during flowering.

Relative transcript level of *GI* **(A)** and *CO* **(B)**, assessed by RT-qPCR, in rosette leaves at different time points after flowering. Error bars indicate standard deviation from the average of biological replicates (n=3). Transcript levels were normalised to *ACT1* and then to then to maximum transcript level. Different letters indicate statistical differences (ANOVA, Tukey's HSD test, p<0.05).

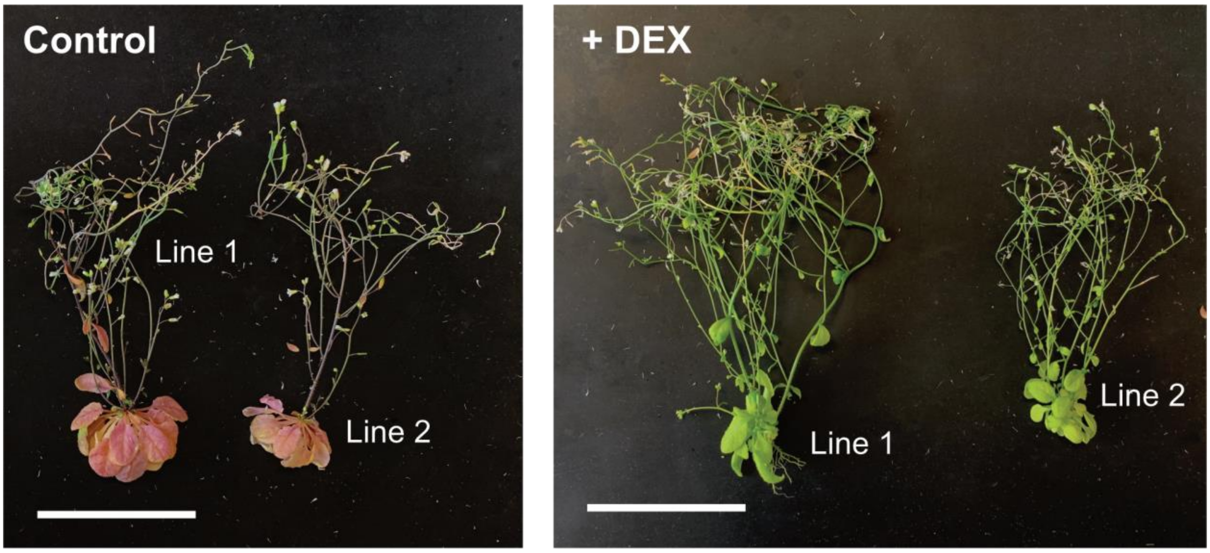

Supplementary Figure S7 | Phenotype of the *35S:FT-GR* lines.

Photograph showing the end-of-flowering phenotype of *35S:FT-GR* lines with no dexamethasone (DEX) in the media. The scale bar represents 5 cm.

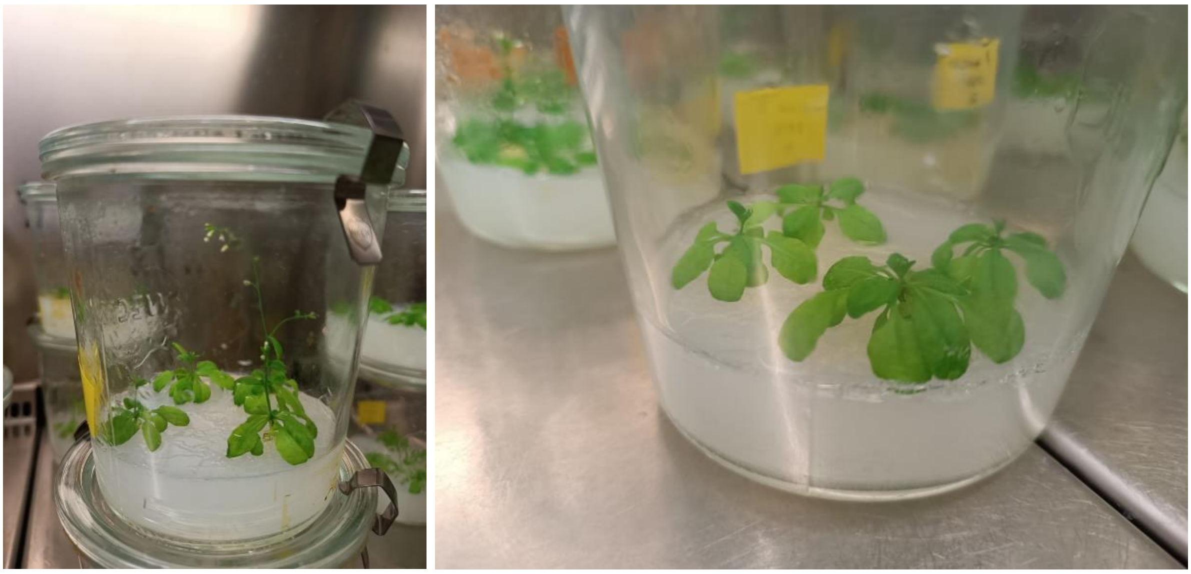

Supplementary Figure S8 | Photographs illustrating the *in vitro* setup for the characterization of the *35S:FT-GR* lines.

Supplementary Table S1 | Description of environmental setups used for Figure 4.

Code	Equipment	Day-time temp	Night-time temp	Day length	Light intensity	Details
A	Grobotic Systems ®	17ºC	15ºC	16 h	100 µmol s^{-1} m^{-2}	Temperature held constantly at 16ºC during flowering
B	Grobotic Systems ®	20ºC	18ºC	16 h	100 µmol s^{-1} m^{-2}	Temperature held constantly at 20ºC during flowering
C	Grobotic Systems ®	22ºC	20ºC	16 h	100 µmol s^{-1} m^{-2}	Temperature held constantly at 22ºC during flowering
D	Grobotic Systems ®	27ºC	25ºC	16 h	100 µmol s^{-1} m^{-2}	Temperature held constantly at 27ºC during flowering
E	Sanyo walk-in room	22ºC	22ºC	Variable	120 µmol s^{-1} m^{-2}	Plants grown at 16 h day length and moved to 8 h day length at bolting
F	Sanyo walk-in room	22ºC	22ºC	Variable	120 µmol s^{-1} m^{-2}	Plants grown at 16 h day length and moved to 8 h at bolting
G	Sanyo walk-in room	22ºC	22ºC	Variable	120 µmol s^{-1} m^{-2}	Plants grown at 16 h day length and moved to 8 h after 4 days of flowering
H	Sanyo walk-in room	22ºC	22ºC	Variable	120 µmol s^{-1} m^{-2}	Plants grown at 16 h day length and moved to 8 h after 8 days of flowering
I	Sanyo walk-in room	22ºC	22ºC	Variable	120 µmol s^{-1} m^{-2}	Plants grown at 16 h day length and moved to 8 h after 12 days of flowering
J	Sanyo walk-in room	22ºC	22ºC	Variable	120 µmol s^{-1} m^{-2}	Plants grown at 16 h day length and moved to 8 h after 16 days of flowering
K	Greenhouse	Variable	Variable	16 h	Variable	Plants grown in 2020, 2021 and 2021 respectively, in a glasshouse where temp was recorded weekly
L	Greenhouse	Variable	Variable	16 h	Variable	
M	Greenhouse	Variable	Variable	16 h	Variable	

Supplementary Table S2 | Primers used for cloning of *35S:FT-GR.*

Name	Sequence
**pro35S attB5r R**	GGGGACAACTTTTGTATACAAAGTTGTTGTCCTCTCCAAATGAAATGAACT
**pro35S attB4 R**	GGGGACAACTTTGTATAGAAAAGTTGGGTGTGTCCTCTCCAAATGAAATGAACT
**FT attB5 F**	GGGGACAACTTTGTATACAAAAGTTGGTATGTCTATAAATATAAGAGACCCTCT
**FT attB2 R**	GGGGACCACTTTGTACAAGAAAGCTGGGTAAAGTCTTCTTCCTCCGCAG

Supplementary Table S3 | Primers used for RT-qPCR.

Name	Sequence
**ACT1 QPCR F**	CGCCGACAGAATGAGCAAAG
**ACT1 QPCR R**	TGCCTTTGCGATCCACATCT
**CO QPCR F**	CTACAACGACAATGGTTCCATTAAC
**CO QPCR R**	CAGGGTCAGGTTGTTGC
**FT QPCR F**	CAACCCTCACCTCCGAGAATAT
**FT QPCR R**	TGCCAAAGGTTGTTCCAGTTGT
**GI QPCR F**	GGGTAAATATGCTGCTGGAGA
**GI QPCR R**	CAGTATGACACCAGCTCCATT
**UBC9 QPCR F**	AGCAATGGAAGCATCTGCCT
**UBC9 QPCR R**	CTTTTGGGTCCAGGTCCGAG
